# Supplementary material for: Transcriptomic Insights and the Development of Microsatellite Markers to Assess Genetic Diversity in the Broodstock Management of Litopenaeus stylirostris
Source: Animals (Basel). 2024 Jun 5;14(11):1685. doi: 10.3390/ani14111685 (PMC11171113; doi:10.3390/ani14111685)
Supplement: Supplementary file 1 [file animals-14-01685-s001.zip › Table S1.pdf]

Table S1. Statistics of the amount of information in the next-generation sequencing of the transcriptome of *Litopenaeus stylirostris*

Sample	Clean Reads (M)	Clean Bases (G)	Q_{20} (%)	Q_{30} (%)	GC (%)	Read Length (bp)
MU1	53.5193	8.0279	97.60	93.37	52.32	150
MU2	52.3542	7.8531	97.62	93.40	51.95	150
MU3	50.4630	7.5695	97.50	93.16	52.11	150
MU4	52.6199	7.8930	97.74	93.66	52.72	150
MU5	44.6145	6.6922	97.68	93.53	52.46	150
MU6	55.5258	8.3289	97.60	93.39	52.04	150
HE1	50.8191	7.6229	98.03	94.30	50.27	150
HE2	49.5993	7.4399	98.10	94.51	50.43	150
LY1	48.4536	7.2680	97.45	92.98	45.75	150
LY2	51.0697	7.6605	97.41	92.91	45.96	150
LY3	37.1968	5.5795	97.55	93.25	45.33	150
LY4	44.4893	6.6734	98.41	95.24	45.25	150
IN1	49.5868	7.4380	97.40	93.01	48.14	150
IN2	46.7253	7.0088	97.76	93.81	47.03	150
Mean	44.7301	6.7095	97.85	93.92	48.94	150

Muscle (MU), hepatopancreas (HE), lymphoid (LY), intestine (IN), and other tissues of 3–4-month-old *Litopenaeus stylirostris* were collected, with three individuals pooled into one tube for each tissue. Total RNA samples were subjected to transcriptome sequencing. Clean Reads refers to the number of reads after low-quality sequencing results are removed, while Clean Bases refers to the number of bases after low-quality sequencing results are removed. Q_{20} and Q_{30} refer to the proportion of reads with a sequencing error rate of less than 1% and 0.1% for this sample, respectively. GC is the ratio of G and C bases in the sample, while Read Length refers to the length of each sequencing sequence measured in base pairs.
