# Supplementary material for: Transcriptomic Insights and the Development of Microsatellite Markers to Assess Genetic Diversity in the Broodstock Management of Litopenaeus stylirostris
Source: Animals (Basel). 2024 Jun 5;14(11):1685. doi: 10.3390/ani14111685 (PMC11171113; doi:10.3390/ani14111685)
Supplement: Supplementary file 1 [file animals-14-01685-s001.zip › Table S2.pdf]

Table S2. Assembly quality statistics of next-generation sequencing of *Litopenaeus stylirostris* transcriptomes

Sample	Total Number	Total Length (nt)	Mean Length (nt)	N50	N70	N90	GC(%)
MU1	25,127	22,592,351	899	2135	943	301	45.23
MU2	21,190	17,924,493	845	1873	855	294	44.13
MU3	23,036	19,878,680	862	1981	906	291	44.22
MU4	22,842	19,320,721	845	1921	864	288	45.38
MU5	21,935	18,630,477	849	1989	867	287	42.54
MU6	26,387	22,995,699	871	2054	905	293	42.54
HE1	33,100	31,185,911	942	1881	1045	344	43.93
HE2	30,544	28,726,202	940	1921	1047	342	43.99
LY1	35,344	47,470,313	1343	2920	1719	527	42.84
LY2	38,532	51,132,776	1327	2889	1699	519	43.17
LY3	39,242	48,827,294	1244	2766	1564	470	42.54
LY4	35,478	45,302,242	1276	2821	1640	488	42.54
IN1	48,558	53,501,938	1101	2470	1321	395	44.51
IN2	40,912	43,818,922	1071	2314	1274	389	43.63
Mean	29,176.80	31,015,286.60	1017.00	2310.20	1,168.00	365.20	42.54

Tissues from *Litopenaeus stylirostris* aged 3–4 months, including muscle (MU), hepatopancreas (HE), lymphoid (LY), and intestine (IN), were collected. Each tissue contained three individuals mixed into one tube, and total RNA samples were subjected to transcriptome sequencing. Total Number refers to the total number of assembled contig or unigene, Total Length refers to the total number of bases of assembled contig or unigene, and Mean Length is the average number of bases of assembled contig or unigene. N50, N70, and N90 are the size of the last contig or unigene fragment when the total length reaches 50%, 70%, and 90%, respectively, in order from smallest to largest; GC is the ratio of bases G and C in the sample.
