# Supplementary material for: Transcriptomic Insights and the Development of Microsatellite Markers to Assess Genetic Diversity in the Broodstock Management of Litopenaeus stylirostris
Source: Animals (Basel). 2024 Jun 5;14(11):1685. doi: 10.3390/ani14111685 (PMC11171113; doi:10.3390/ani14111685)
Supplement: Supplementary file 1 [file animals-14-01685-s001.zip › Table S3.pdf]

Table S3. Quality statistics of unigenes assembly for next-generation sequencing of *Litopenaeus stylirostris* transcriptomes

Sample	Total Number	Total Length (nt)	Mean Length (nt)	N50	N70	N90	GC (%)
MU1	19,463	20,140,617	1034	2164	1022	359	44.98
MU2	16,456	16,125,928	979	1923	924	352	44.02
MU3	17,873	17,857,965	999	2012	977	347	44.08
MU4	17,855	17,208,240	963	1931	914	339	45.18
MU5	17,093	16,800,715	982	2024	944	341	44.83
MU6	20,508	20,716,246	1010	2109	981	352	44.43
HE1	26,656	28,418,019	1066	1936	1114	402	43.82
HE2	24,193	25,930,710	1071	1980	1116	403	43.87
LY1	28,716	43,089,196	1500	2949	1760	595	42.80
LY2	31,216	46,252,192	1481	2918	1747	585	43.16
LY3	31,574	44,253,487	1401	2816	1621	541	43.48
LY4	28,649	41,140,287	1436	2865	1692	559	42.52
IN1	38,646	48,392,997	1252	2539	1396	459	44.42
IN2	32,782	39,577,614	1207	2365	1337	448	43.56
All-Unigene	53,263	94,147,609	1767	3478	2121	773	44.56

RNA samples were collected from different tissues, including muscle (MU), hepatopancreas (HE), lymphoid (LY), intestinal tract (IN), and other tissues of 3–4-month-old *L. stylirostris*. Three biological replicates were mixed in one tube, and transcriptome sequencing was performed. The table shows the results of transcriptome assembly, including the total number, total length, and mean length of the assembled contigs or unigenes; N50, N70, and N90 (the length of the last contig or unigene to reach 50%, 70%, and 90%, respectively, of the total length, when all contigs or unigenes are arranged in order from small to large), and GC content (the ratio of bases G and C in the sample).
