# Supplementary material for: Transcriptomic Insights and the Development of Microsatellite Markers to Assess Genetic Diversity in the Broodstock Management of Litopenaeus stylirostris
Source: Animals (Basel). 2024 Jun 5;14(11):1685. doi: 10.3390/ani14111685 (PMC11171113; doi:10.3390/ani14111685)
Supplement: Supplementary file 1 [file animals-14-01685-s001.zip › Table S4.pdf]

Table S4. Functional annotation of the *Litopenaeus stylirostris* transcriptome dataset against publicly available databases

Transcriptome Dataset[a]	Unigene Number	Percentage
NR	22,539	42.32%
NT	8122	15.25%
Swiss-Prot	21,026	39.48%
COG	7978	14.98%
GO	8828	16.57%
KEGG	18,565	34.86%
Overall (total annotation)[b]	24,066	45.18%
Total	53,263	100%

[a]NR: Unigenes with NCBI non-redundant protein; NT: Nucleotide Database; Swiss-Prot: A curated protein sequence database that strives to provide high levels of annotation; COG: Clusters of Orthologous Groups; GO: Gene Ontology; KEGG: Kyoto Encyclopedia of Genes and Genomes. [b]Number of unigenes annotated in at least one functional database.
