# Supplementary material for: Transcriptomic Insights and the Development of Microsatellite Markers to Assess Genetic Diversity in the Broodstock Management of Litopenaeus stylirostris
Source: Animals (Basel). 2024 Jun 5;14(11):1685. doi: 10.3390/ani14111685 (PMC11171113; doi:10.3390/ani14111685)
Supplement: Supplementary file 1 [file animals-14-01685-s001.zip › Table S5.pdf]

Table S5. Statistics of prediction results of unigene coding sequence regions in the *Litopenaeus stylirostris* transcriptome

Parameters	BLAST	ESTScan	Total
Total Number	23,635	7006	30,641
Total Length	29,647,231	5,486,904	35,134,135
Mean Length	1254	783	1146
N50	1816	1095	1693
N70	1186	678	1087
N90	601	336	532
GC (%)	51.32	54.07	51.75

The coding sequence regions of the unigenes were compared using two software tools, BLAST and ESTScan. Total Number: number of unigenes that were successfully compared; Total Length: number of bases of the unigenes that were successfully compared; Mean Length: average number of bases in the unigenes that were successfully compared; N50, N70 and N90: size of the last contig or unigene fragment to reach 50%, 70% and 90% of the total length, respectively, when all contigs or unigenes are arranged from small to large; GC: ratio of G and C bases to total nucleotides.
