# Supplementary material for: Transcriptomic Insights and the Development of Microsatellite Markers to Assess Genetic Diversity in the Broodstock Management of Litopenaeus stylirostris
Source: Animals (Basel). 2024 Jun 5;14(11):1685. doi: 10.3390/ani14111685 (PMC11171113; doi:10.3390/ani14111685)
Supplement: Supplementary file 1 [file animals-14-01685-s001.zip › Table S7.pdf]

Table S7. Primer pairs designed from the NCBI literature database and available for genetic polymorphism testing of broodstock in *Litopenaeus stylirostris*

Locus (n=58)	Motif	Primer (5'→3')	Size (bp)
LV1	$(CT)_{14}$	F: CCACCTAACACTGAAATGCAAG	237
		R: GGGAGAGAGAGAGAGAGAGAGAGA	
LV2	$(AG)_{9}$	F: GTGTGTGATGGTGTGTGTTTTG	108
		R: TGATGTGCAGGTTTCAGTCTTT	
LV3	$(AG)_{9}$	F: ATGATGACCAAAGGATATTGGC	397
		R: GACCAGCAGTTCAAAGGGATT	
LV4	$(CA)_{57}$	F: TTTTACTCTTCGTAAGCGCGAG	331
		R: CACCAACGCCATCTATTATTCC	
LV5	$(CT)_{18}$	F: TGTGTGTGTGTCTTCCTCTTCC	166
		R: CGCTATTTAAGCGAGTGTGTGT	
LV6	$(TA)_{23}$	F: TTCAACTAACTCACGGCCATACT	128
		R: CGTTTCTTGGTTCCTTAATCCA	
LV7	$(AT)_{23}$	F: TATGAATACTGATGCACACGCA	307
		R: TAACAATACAAACGCACACACG	
LV8	$(TTTCTC)_{6}$	F: GGATGAATGGAGAGCGAAATA	266
		R: AAAGAGAAAGAGAGAGAGAGAGAGAGA	
LV9	$(ACTC)_{21}$	F: TCTCCAGCCTAAACATGGGTAT	832
		R: TTTGTGTGTGTGTGTGTGTGTG	
LV10	$(CTCA)_{6}$	F: CGTGGTTAAGTGATTGATGTGG	370
		R: AGCTTCCAGTCTCCAGTTTCC	
LV11	$(CTAAC)_{7}$	F: GTAACCTAACCTAACCTCCCCA	225
		R: GTAAATCACTCTAATCCACGATAGG	
LV12	$(TA)_{24}$	F: TCGTATTCTGTGACTTCGCATC	391
		R: TGAGGGAGAGAGAGAGAGAGGA	
LV13	$(CA)_{16}$	F: GGGGTCTGAGTTCTGTAGTGGA	275
		R: ATCATCACGGAATGTAAAAGGG	
LV14	$(TAT)_{6}$	F: TTGGTCGGATAACCTATTTTGC	322
		R: TTTTCATGCGCTATTGTGGAC	
LV15	$(TTC)_{9}$	F: AATGGGTCAGCATCAAACATC	400
		R: GGGAGTAGGAGGAGGAGGAG	
LV16	$(TC)_{17}$	F: GCAAGCATTTTGGTTACTCCC	194
		R: CACACACACACACACACACACA	
LV17	$(CCTAA)_{6}$	F: CGTGGATTAGAGTGATTTACGAAC	143
		R: CAGTACCTGCAATTTAGGGAGG	
LV18	$(CA)_{35}$	F: TCTAAACCACCCACCCTCATAC	321
		R: GAAAATGGGGAAGATGAATGG	
LV19	$(TC)_{11}$	F: AAATTCGGTGTGGTTGCTAATC	136
		R: CATGTGTAGATACGTTTTGCCG	
LV20	$(CA)_{29}$	F: TTGACAGCTTGGCGAATACTAC	399
		R: ATGTGCGTGTGTATGTGTGTGT	
LV21	$(CA)_{19}$	F: GAGGCACGAGACACATACAGAC	183

		R: GTGTAGGGTAGGGGTGAGAGTG	
LV22	$(AC)_{31}$	F: TTCACACACACACACACACACA	233
		R: ATAAGTGTATTGGGGAGGGGC	
LV23	$(CA)_{40}$	F: CAGGTACACACACACATTCACG	272
		R: GTCTCGGTTTTCTTCCCAGTC	
LV24	$(AC)_{31}$	F: TGCAAATATCTCGTGTCTCTCA	384
		R: TGTGCTCGTGTGTTTATGAGTT	
LV25	$(AT)_{27}$	F: ATATTCGTGTGTTTTCTTGCCC	312
		R: TCTAGTTTGTGCATTGTGGGTT	
LV26	$(TGTCTC)_{10}$	F: ATGTGAAGACCTCGTTACTCCC	362
		R: GTAGAGATGATAAGAACCCGGC	
LV27	$(TC)_{14}$	F: AACAGCCAGTCAACCGATTATT	274
		R: GGGAGAGAAAGAAAGGGATAGG	
LV28	$(TC)_{44}$	F: GTCCCATTCCCAATTCAGATAA	217
		R: TTCCACATTTCCTCCTTCTAGC	
LV29	$(TAA)_{12}$	F: CAAAAGTTGGCACGGGAA	160
		R: GACTCACATCCTCATCCTCATTATT	
LV30	$(AC)_{34}$	F: CAAACACACACACACACACACA	240
		R: TCTTCATTTCTGTACTTCTGTCCG	
CNM1	$(AAAAT)_{4}$	F: GAGCGTATCTAACCTCA	284
		R: TATGGCTATTGTAACTCTTC	
CNM2	$(AAAAT)_{3}$	F: GCTGAAACGCTCGTCA	265
		R: TGGGCATACTGGGAAA	
CNM3	$(ATTTT)_{4}$	F: AAACCACCCTGACCATC	284
		R: CTGTGCCAAATTACAAGC	
CNM4	$(AAAAC)_{3}$	F: CGAGCACAGGAAGATA	257
		R: TCTGGGAGAAGGGATA	
CNM5	$(ATTTT)_{3}$	F: ACAGGGCAGGACAATA	237
		R: GTTAACTGAGCCATACTTT	
CNM6	$(GCTC)_{4}$	F: TCACCATCGCCAGAAA	115
		R: CCAGGGAAGAGGAGGA	
CNM7	$(GCAA)_{4}$	F: CACAGTGCGAGATGGC	169
		R: TACCGAACAAGGAATACAAT	
CNM8	$(AAAT)_{4}$	F: ACAACTTCAAAGCTACA	252
		R: TCAGTCTCATCTTCCAT	
CNM9	$(TTTG)_{4}$	F: TGCTCAAGTCGTTACT	116
		R: GAGGTTTCTGTTCTATAA	
CNM10	$(CTTC)_{4}$	F: CTTCATACCCATTCTTTCT	300
		R: GCAATAGGCTACAGTTCC	
CNM11	$(TTGT)_{4}$	F: GACATGAGGTATAGCCATTA	208
		R: TATGCACCCTGCTGAC	
CNM12	$(TAAA)_{4}$	F: TTTGATGGGCAAGGAG	257
		R: AGTGGAGTGGCTGGAA	
CNM13	$(AAAG)_{4}$	F: TAGAAGGCAAAGCAGT	275
		R: ATTCTATCACCACCGT	
CNM14	$(AAAC)_{3}$	F: GTTCTCGAACATGGGA	295

		R: GGGTGATGCAACCTAT	
CNM15	$(GATT)_3$	F: TGACAGTAACTCCCAAAT	195
		R: GAATGCAGGAAACATG	
CNM16	$(ATG)_6$	F: AGATACGCTTCCTAATGAT	157
		R: GCTAGTTCCTGCTCCC	
CNM17	$(AGC)_6$	F: AACGCAGCGCAGAAGA	268
		R: TGGAATTGTGAGCGGATA	
CNM18	$(CAA)_6$	F: CAACCAGGAAATAGAACAG	135
		R: GCAGCCTTACCACGAC	
CNM19	$(GGC)_6$	F: GACAGACAGTGGTGGCG	297
		R: CGTTCTCCTTGCGTGATG	
CNM20	$(AAT)_5$	F: TTTCTGCCACGGAGTT	144
		R: CTGTTGCCCAAATAGC	
CNM21	$(TTA)_5$	F: AAGTCCAGACAACGAG	256
		R: TAACCTTTAGCAACCT	
CNM22	$(TGA)_5$	F: CGTAAGATGTGCCAGT	248
		R: CAGTTATAAAGTCAAAAGTA	
CNM23	$(TTA)_5$	F: TGGATTTGCCGATTGA	252
		R: TCCCAGCACTTGTCATC	
CNM24	$(TGG)_5$	F: TTGCTGCTTACTGTCTTGC	296
		R: ATCATCCGACTCTTCCTCT	
CNM25	$(CAC)_5$	F: GTTCTGTTTACAATTGGTTC	206
		R: GAGGAGGACTGAGGGTG	
CNM26	$(ATT)_5$	F: TGAAGCCATTGTCTGT	263
		R: TTGTGGGAGATGATGC	
CNM27	$(TGG)_5$	F: TTGCTGCTTACTGTCTTGC	297
		R: CATCATCCGACTCTTCCT	
CNM28	$(GTT)_4$	F: CGTACAAGGCATTGGG	278
		R: GCATCTACTTTGACGCACT	
