# Supplementary figures and images for: Transcriptomic Insights and the Development of Microsatellite Markers to Assess Genetic Diversity in the Broodstock Management of Litopenaeus stylirostris
Source: Animals (Basel). 2024 Jun 5;14(11):1685. doi: 10.3390/ani14111685 (PMC11171113; doi:10.3390/ani14111685)

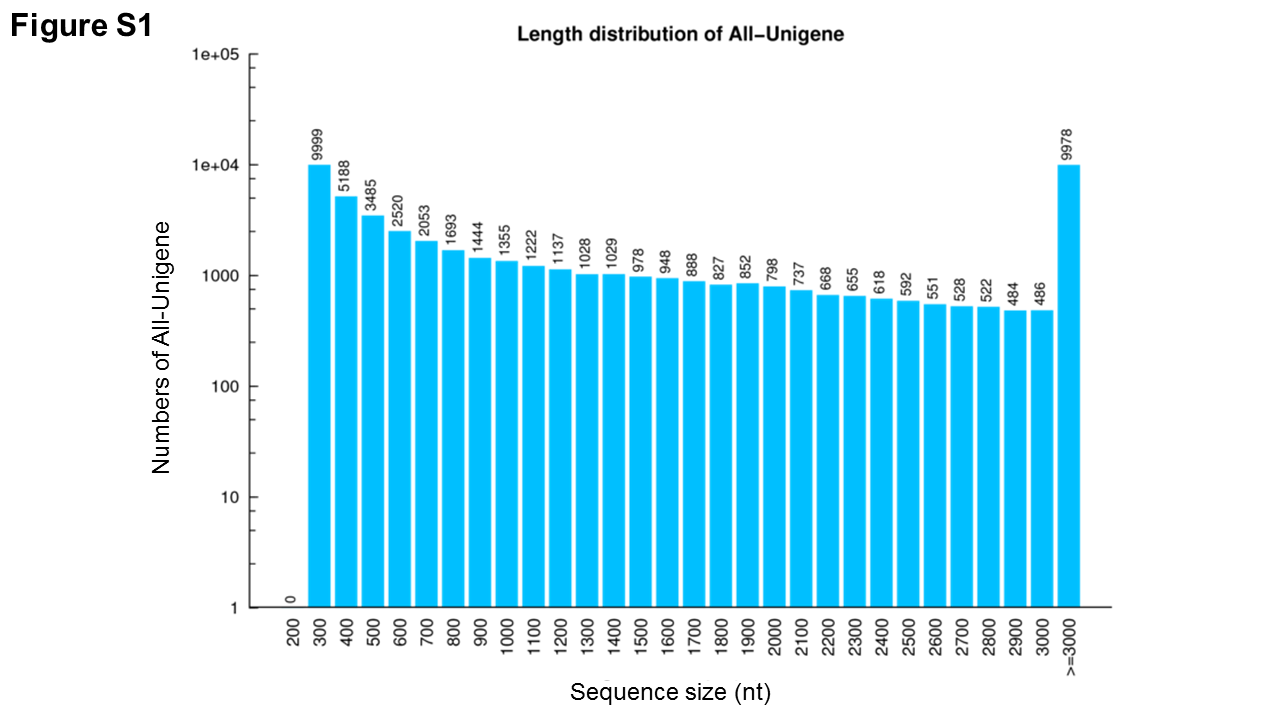

Supplement: Supplementary file 1 [file animals-14-01685-s001.zip › Supplementary Figure_S1.tif]

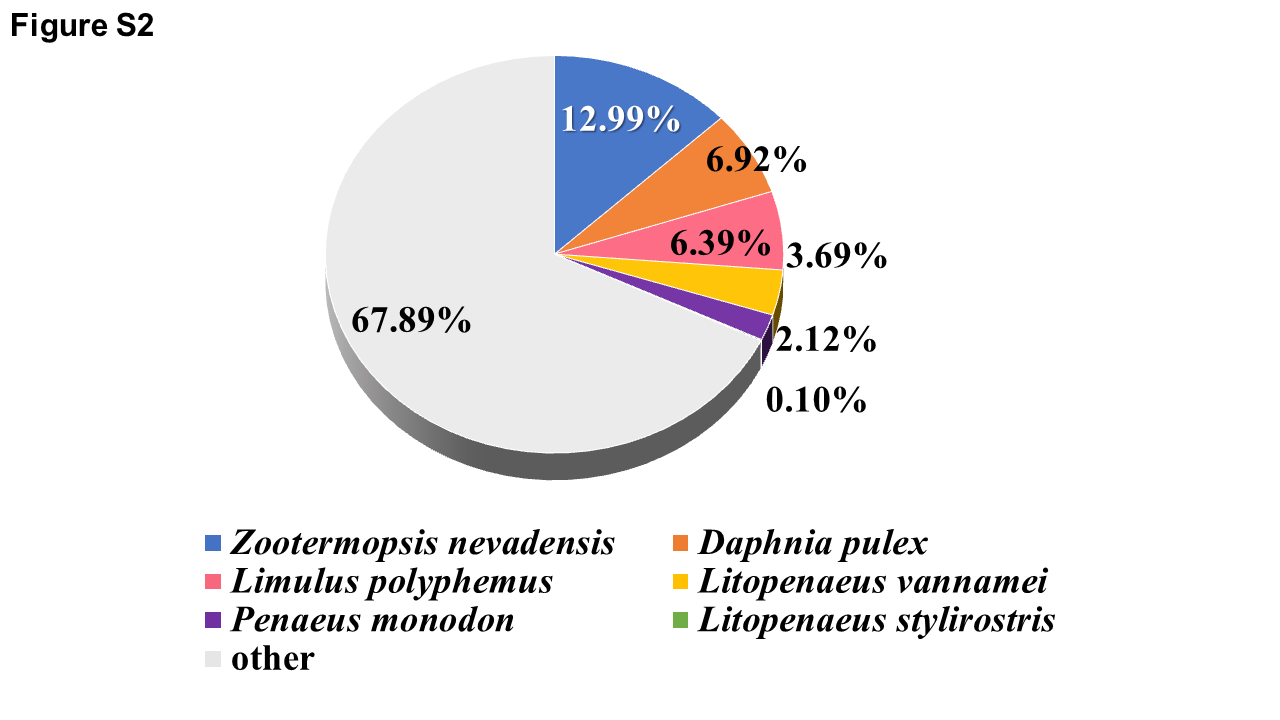

Supplement: Supplementary file 1 [file animals-14-01685-s001.zip › Supplementary Figure_S2.tif]

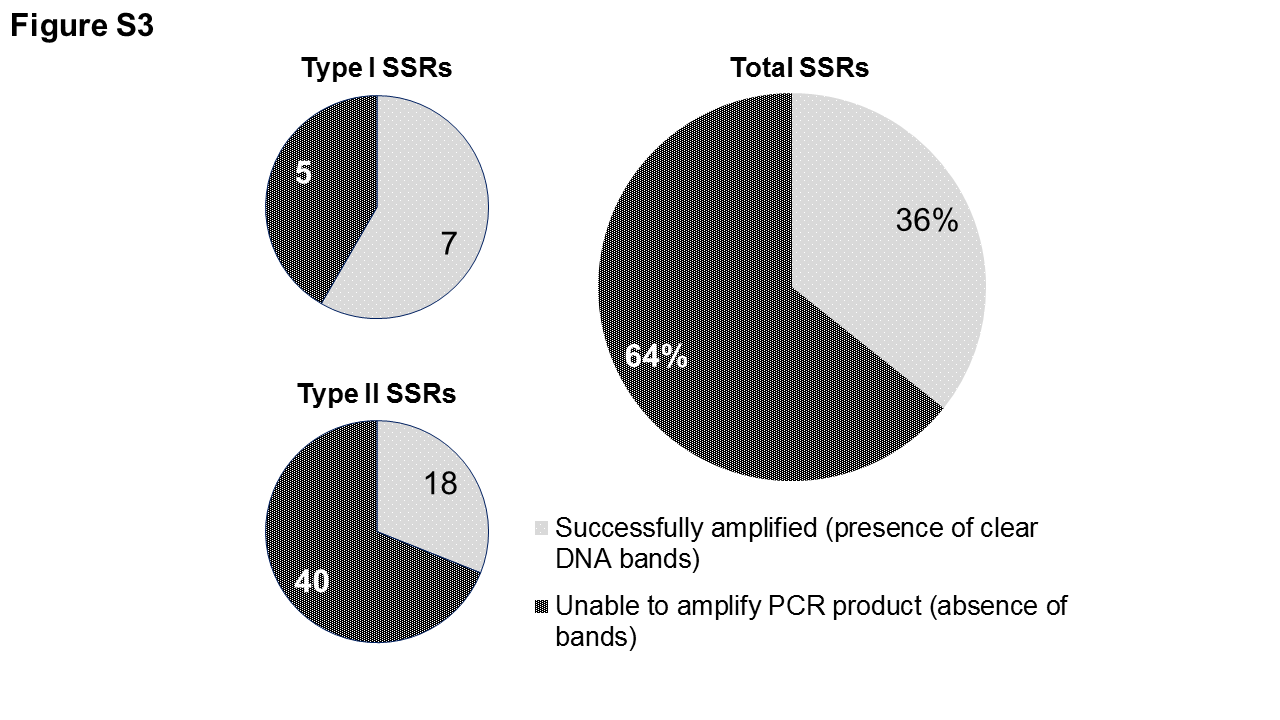

Supplement: Supplementary file 1 [file animals-14-01685-s001.zip › Supplementary Figure_S3.tif]
